# Demographic toxicology of insect growth regulators on the nontarget ectolarval parasitoid *Habrobracon hebetor*

**DOI:** 10.1038/s41598-024-75634-4

**Published:** 2024-10-31

**Authors:** Amany N. Mansour, Karem S. Ghoneim, Khaled S. Hamadah, Ahmed A. Abo Elsoud

**Affiliations:** 1 Plant Protection Department, Desert Research Center, Cairo, Egypt; 2 Department of Zoology and Entomology, Faculty of Science, Al-Azhar University, Cairo, Egypt

**Keywords:** Entomology, Agroecology, Plant sciences

## Abstract

*Habrobracon hebetor* Say (Hymenoptera: Braconidae) is one of the most important parasitoids of many pyralid moths, including the olive leaf moth, *Palpita unionalis* Hubner (Lepidoptera: Pyralidae). The widespread use of insecticides threatens natural enemies. Assessing the side effects of insecticides on nontarget organisms supports the rational use of insecticides during field application. The present study evaluates the lethal and sublethal effects of three insect growth regulators (IGRs), novaluron, methoxyfenozide, and pyriproxyfen, on *P. unionalis* and the demographic toxicology of these IGRs on its parasitoid *H. hebetor*. The LC_50_ values of these IGRs on *P. unionalis* were 0.97, 0.176, and 0.00009 ppm, respectively, indicating that pyriproxyfen was the most toxic. When *H. hebetor* adults were exposed to these LC_50_ levels under laboratory conditions to determine possible side effects. The IGRs did not affect the paralysis and parasitism rates of the parasitoid nor the sex ratio of its offspring. IGR treatments slightly reduced hatching rates and immature survival by 15–25%, indicating moderate effects on the early developmental stages of *H. hebetor*. The longevity and fecundity of treated females were each reduced to < 50% of their respective values in untreated females. Additionally, some demographic parameters of the parasitoid were significantly affected by the IGRs. Nevertheless, despite these observed effects, the positive net reproductive rate (R_0_ > 0) and intrinsic rate of increase (r_m_ > 1) of *H. hebetor* indicated an exponential population increase that reflects the compatibility of the IGRs with the parasitoid. Our results demonstrated that the tested IGRs could be categorized as relatively harmless compounds to the parasitoid. Following these laboratory assessments, field studies will be required to confirm the effects of the tested IGRs on *H. hebetor* as well as other nontarget organisms.

## Introduction

In recent decades, the growing human population has led to agricultural expansion, which, along with climate change, has resulted in a rise in insect pest populations. This increase in pest activity has necessitated the extensive use of chemical pesticides. However, the widespread use of these pesticides has had significant environmental consequences, including the disruption of ecosystems and the detrimental impact on natural enemies that play a crucial role in pest control​^1,2^.

When an insecticide is found to have potential effects against pest species, it is pertinent to determine its side effects on associated natural enemies^3^. This is for the reason that various studies have proven that exposure to low doses of different insecticides can affect the reproduction of natural enemies^4–6^. For effective IPM programs, the applied pesticides must be compatible with natural enemies^7,8^. Insect growth regulators (IGRs) are novel insecticides that can affect insect moulting and metamorphosis with better ecotoxicological profile than conventional pesticides^9,10^. IGRs can have contact effects on natural enemies over the initial hours following insecticide application; however, they are generally assumed to be less harmful when compared with other synthetic chemical insecticides^11^. Novaluron is a benzoylphenyl urea insecticide that suppresses endocuticular deposition and moulting by inhibiting chitin formation^12^; it is known to have low toxicity in birds and humans^13^. Methoxyfenozide is a diacylhydrazine insecticide that mimics the moulting hormone and binds to the ecdysone receptor complex in lepidopterans; it has an excellent safety margin to a wide range of beneficial insects^14^. Pyriproxyfen is a juvenile hormone analogue that mimics its activities and competes against it for receptor binding sites^15^.

Over recent decades, the olive leaf moth, *Palpita unionalis* has become a pest species of great concern^16,17^. Its importance is due to its effects on young olive trees in nurseries, e.g., stunted growth; moreover, when its population numbers are high, *P. unionalis* can destroy a significant portion of an olive crop^18,19^. The control of *P. unionalis* is based on extensive use of conventional insecticides^20^. The adverse effects of such pesticides can include outbreaks of pests due to the loss of their natural enemies and resistance to agrochemicals^21^. Parasitoids have a very close association with their hosts^22^. Mahdavi, et al.^23^ stated that recommended insecticides should be those that are effective against pests but less noxious to their natural enemies. Furthermore, studying the effects of pesticides on nontarget natural enemies can facilitate the combined use of pesticides with biocontrol agents in integrated pest management (IPM) programs^24,25^.

*Habrobracon hebetor* (= *Bracon hebetor*) is a cosmopolitan larval parasitoid widely recognized for its role in controlling various pyralid lepidopteran pests, including *P. unionalis* in olive orchards^26^. Previous studies have evaluated the side effects of several compounds on *H. hebetor* to assess their suitability for IPM^27–29^, many studies have utilized both field and laboratory factitious hosts in pesticide toxicity experiments. For instance, Abedi, et al.^30^ evaluated the effect of pyridalil and methoxyfenozide on the functional response of *H. hebetor* as an important parasitoid of *Helicoverpa armigera* Hubner (Lepidoptera: Noctuidae) when using different larval densities of the factitious host *Ephestia kuehniella* Zell. (Lepidoptera: Pyralidae). Additionally, Blibech, et al.^31^ studied the direct effect of deltamethrin and spinosad for use against *Prays oleae* Bern. (Lepidoptera: Hyponomeutidae), a devastating olive pest, on different *Trichogramma* species (Hymenoptera: Trichogrammatidea) in eggs of the factitious host *E. kuehniella*.

Although IGRs have low toxicity against mammals and are considered compatible with natural enemies^32^, lethal effects have been recorded against some biocontrol agents^21,33,34^. Thus, IGRs must be evaluated for their compatibility with natural enemies. Hence, the present study was designed to evaluate the effects of three IGRs, namely novaluron (a chitin synthesis inhibitor), methoxyfenozide (a molting hormone agonist), and pyriproxyfen (a juvenile hormone analog), on both *P. unionalis* and its associated parasitoid *H. hebetor*. By elucidating the impacts of these IGRs on the target pest and its parasitoid, this research will contribute to evaluating the feasibility and ecological implications of utilizing these IGRs for olive leaf moth control in orchard environments.

## Materials and methods

### Experimental insects

Specimens of *H. hebetor* were collected from olive orchards in Matrouh Governorate, Egypt, and maintained under laboratory conditions (27 °C ± 2 °C, 65% ± 5% RH, and a 16:8-h (L: D) photoperiod) using the greater wax moth *Galleria mellonella* L. (Lepidoptera: Pyralidae) as a factitious host. The artificial diet used to feed *G. mellonella* larvae was previously described by Bhatnagar and Bareth^35^. *Habrobracon hebetor* was periodically exposed to the larvae of its original host *P. unionalis* (reared on fresh olive leaves under the abovementioned conditions) and always supplied with honey droplets for the nourishment of adult wasps^36^. All experimental research studies involving olive plants and insects were performed in accordance with the relevant institutional, national, and international guidelines and legislation. No endangered or protected species were involved in the study.

### IGR preparation and bioassay

To determine the lethal and sublethal effects of the tested IGRs, a concentration series of each IGR was prepared via dilution with distilled water as follows: novaluron at 100.0, 10.0, 1.0, 0.1, 0.01, and 0.001 ppm; methoxyfenozide at 100.0, 10.0, 1.0, 0.1, 0.01, and 0.001 ppm; and pyriproxyfen at 1.0, 0.1, 0.01, 0.001, 0.0001, and 0.00001 ppm. Ten replicates of fresh olive twigs were dipped in each concentration of each IGR for 5 min and then air dried. Three *P. unionalis* larvae (10 days old) were added to each twig and left for 24 h. Control larvae were added to water-treated olive twigs. The larvae were subsequently provided with fresh untreated olive leaves daily and observed until adult emergence. All mortalities of larvae, pupae, and adults were recorded daily and corrected according to Abbott’s formula^37^. The LC_50_ values of the IGRs were calculated using probit analysis according to the method of Finney^38^.

### Compatibility of the tested IGRs with *Habrobracon hebetor*

To assess the compatibility of the tested IGRs with *H. hebetor*, five young (< 24 h old) mated female parasitoids were exposed to freshly prepared IGR residues in Petri dishes. The Petri dishes (5.5 × 1.4 cm) were sprayed with 3 ml of the LC_50_ of the tested IGRs before they were hand-rolled and left to air dry. This method is consistent with the approach used by Saber and Abedi^39^ and Dakhli, et al.^40^, who have demonstrated its effectiveness in similar toxicological studies. Six Petri dishes were used for each IGR. After 24 h, each living female was transferred to a clean Petri dish and provided with honey droplets daily as food and with one *G. mellonella* larva that served as a host for progeny development. This process was repeated using untreated Petri dishes for the purpose of comparison; observations continued until the females died.

For each female, the following observations were recorded: total longevity [mean days per female ± standard error (SE)], fecundity (mean number of eggs per female ± SE), rate of host larval paralysis (number of paralyzed larvae from the total number of larvae introduced), and rate of host larval parasitism (number of parasitized larvae of the total number of larvae introduced). Paralyzed larvae are those that have been immobilized by the parasitoid’s sting but remain alive, whereas parasitized larvae have been both stung and used for oviposition by the parasitoid. Parasitized larvae were kept under the rearing conditions described above and progeny were recorded. Hatchability percentage (number of hatched eggs of the total eggs deposited), rate of larval survival (number of pupae from the number of larvae), rate of pupal survival (number of emerged adults from the number of pupae), and the sex ratio of offspring (females from the total number of offspring) were calculated.

Daily schedules of both mortality and fecundity were integrated into a life table. These data were used to calculate the net reproductive rate (R_0_), mean generation time (T), intrinsic rate of increase (r_m_), finite rate of increase (𝜆), and doubling time (DT).

### Data analysis

Probit analysis was used to calculate the median Lethal Concentration (LC_50_) values with fiducial limits at 95% using the SPSS Statistics program (version 26). The goodness of fit was determined using a χ^2^ test at 5%. Data from the life table were entered into the 48 Basic program to calculate life table parameters^41,42^. For statistical analysis of the data obtained from parasitoid experiments, SPSS was used to apply one-way ANOVA with mean separation at a 5% significance level achieved using Tukey’s test.

### Ethics

According to journal policies involving experimental research and field studies on plants (either cultivated or wild), we declare that our research complies with relevant institutional, national, and international guidelines and legislation. All plant material was collected with the permission of the farms on which we worked, and no indigenous plants or animals were harmed in the process.

### Data availability

The dataset generated during the current study is available from the corresponding author upon request.

## Results

### Bioassay

After 24 h of exposure, IGRs were lethal to *P. unionalis* larvae with LC_50_ values for novaluron, methoxyfenozide, and pyriproxyfen of 0.97, 0.176, and 0.00009 ppm, respectively (Table 1). According to lethal concentrations with 95% confidence limits, the toxicities of the IGRs on *P. unionalis* differed. As shown in Table 1, pyriproxyfen was the most toxic of the IGRs followed by methoxyfenozide and novaluron.

**Table 1:** Toxicity and lethal effects of IGRs (novaluron, methoxyfenozide, and pyriproxyfen) on the olive leaf moth, P. Unionalis.

Insecticide	*N*	Slope ± SE	χ^2^	Lethal concentrations (ppm) (95% CL)*		
				LC_30_	LC_50_	LC_90_
Novaluron	210	0.88 ± 0.09	16.98	0.24 (0.00–1.22)	0.97 (0.06–4.98)	27.86 (5.32–4992.19)
Methoxyfenozide	210	0.73 ± 0.05	24.37	0.03 (0.00–0.15)	0.176 (0.03–0.85)	9.77 (1.71–532.76)
Pyriproxyfen	210	1.04 ± 0.12	12.15	0.00001 (0.00000–0.00070)	0.00009 (0.00000–0.00100)	0.00300 (0.00000–0.08500)

^*^Lethal concentrations and 95%confidence limits (CL) were estimated using probit analysis in SPSS.

The effects of the LC_50_ values of the three tested IGRs on *H. hebetor* are shown in Table 2. The IGRs significantly reduced female longevity and fecundity to almost half of their normal (control) values (longevity: *F* = 27.59; *df* = 3, 96; *P* < 0.001, fecundity: *F* = 45.82; *df* = 3, 93; *P* < 0.001). However, the rates of both paralysis (*F* = 0.35; *df* = 3, 93; *P* = 0.788) and parasitism (*F* = 0.33; *df* = 3, 93; *P* = 0.801). 1) were not affected by the IGRs.

**Table 2:** Direct effects of novaluron, methoxyfenozide, and pyriproxyfen (LC50) on H. hebetor females.

Treatment	Longevity (days)	Fecundity
Novaluron	7.17 ± 0.50^b^*	18.81 ± 1.19^b^
Methoxyfenozide	6.53 ± 0.37^b^	18.87 ± 1.60^b^
Pyriproxyfen	6.50 ± 0.37^b^	20.57 ± 1.71^b^
Control	13.50 ± 0.60^a^	52.30 ± 3.05^a^

^*^Data represented by mean±SE and means in the same column followed by the same letter are not significantly different based on Tukey’s test (SPSS).

**Fig. 1:**
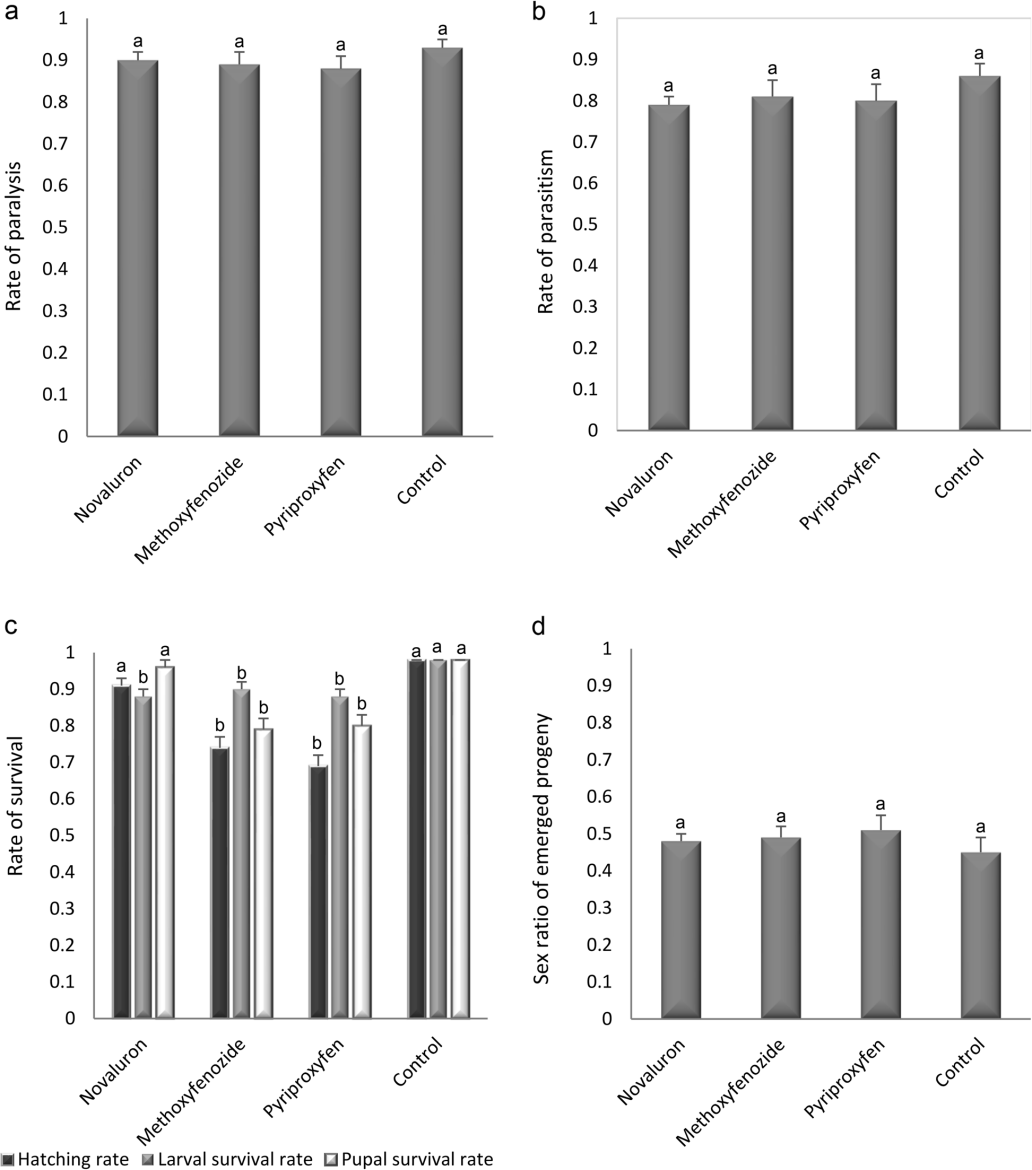
Effects of IGRs on *H. hebetor* and its Progeny. Rate of paralysis (**a**) and parasitism (**b**); rate of survival of eggs, larvae and pupae (**c**) and sex ratio (female/total) of progeny (**d**) of *H. hebetor* females on *G. mellonella* larvae after treated with IGRs.

Figure 1 shows the effects of IGRs that extended to the progeny of *H. hebetor*. Slight reductions in the hatching of parasitoid eggs (*F* = 21.62; *df* = 3, 91; *P* < 0.001) as well as larval (*F* = 2.47; *df* = 3, 91; *P* = 0.043) and pupal survival rates were observed (*F* = 13.04; *df* = 3, 91; *P* < 0.001). The sex ratio of the offspring was not affected by the IGRs (*F* = 0.27; *df* = 3, 91; *P* = 0.842) (Fig. 1).

### Effects of IGRs on the life table parameters of *Habrobracon hebetor*

The effects of LC_50_ levels of the tested IGRs on the demographic parameters of *H. hebetor* are shown in Table 3. The R_0_ of treated wasps was significantly reduced compared with the R_0_ of the control (*F* = 20.84; *df* = 3, 11; *P* < 0.001); the R_0_ of the untreated wasps was 22.28, whereas the R_0_ values of novaluron-, methoxyfenozide-, and pyriproxyfen-treated wasps were 6.79, 5.09, and 5.24, respectively. Similarly, the rm of *H. hebetor* was significantly affected by the IGRs (*F* = 14.39; *df* = 3, 8; *P* < 0.001). Both 𝜆 and T were slightly reduced after IGR treatment (𝜆: *F* = 15.10; *df* = 3, 8; *P* < 0.001. T: *F* = 18.46; *df* = 3, 11; *P* < 0.001). In contrast, the DT of parasitoid populations was increased by the IGRs (*F* = 5.61; *df* = 3, 11; *P* < 0.001); the highest DT was recorded in methoxyfenozide-treated insect populations, whereas the lowest DT was recorded in control populations.

**Table 3:** Effects of the LC50 levels of novaluron, methoxyfenozide, and pyriproxyfen on the life table parameters of H. Hebetor.

Treatment	Net reproductive rate (*R*_0_)**	Intrinsic rate of increase (*r*_m_) (day^−1^)	Finite rate of increase (𝜆) (day^−1^)	Generation time (T) (day)	Doubling time (DT) (day)
Novaluron	6.79 ± 0.24^b^*	0.13 ± 0.00^b^	1.14 ± 0.00^b^	14.48 ± 0.02^b^	5.24 ± 0.08^b^
Methoxyfenozide	5.09 ± 0.35^b^	0.12 ± 0.01^b^	1.13 ± 0.01^b^	13.87 ± 0.06^b^	5.91 ± 0.29^b^
Pyriproxyfen	5.24 ± 0.45^b^	0.12 ± 0.01^b^	1.13 ± 0.01^b^	13.93 ± 0.15^b^	5.83 ± 0.34^b^
Control	22.28 ± 2.41^a^	0.19 ± 0.01^a^	1.21 ± 0.01^a^	16.43 ± 0.27^a^	3.67 ± 0.11^a^

^*^Data represented by mean±SE and means in the same column followed by the same letter are not significantly different based on Tukey’s test (SPSS).

^**^Mean number of female offspring/♀.

**Fig. 2:**
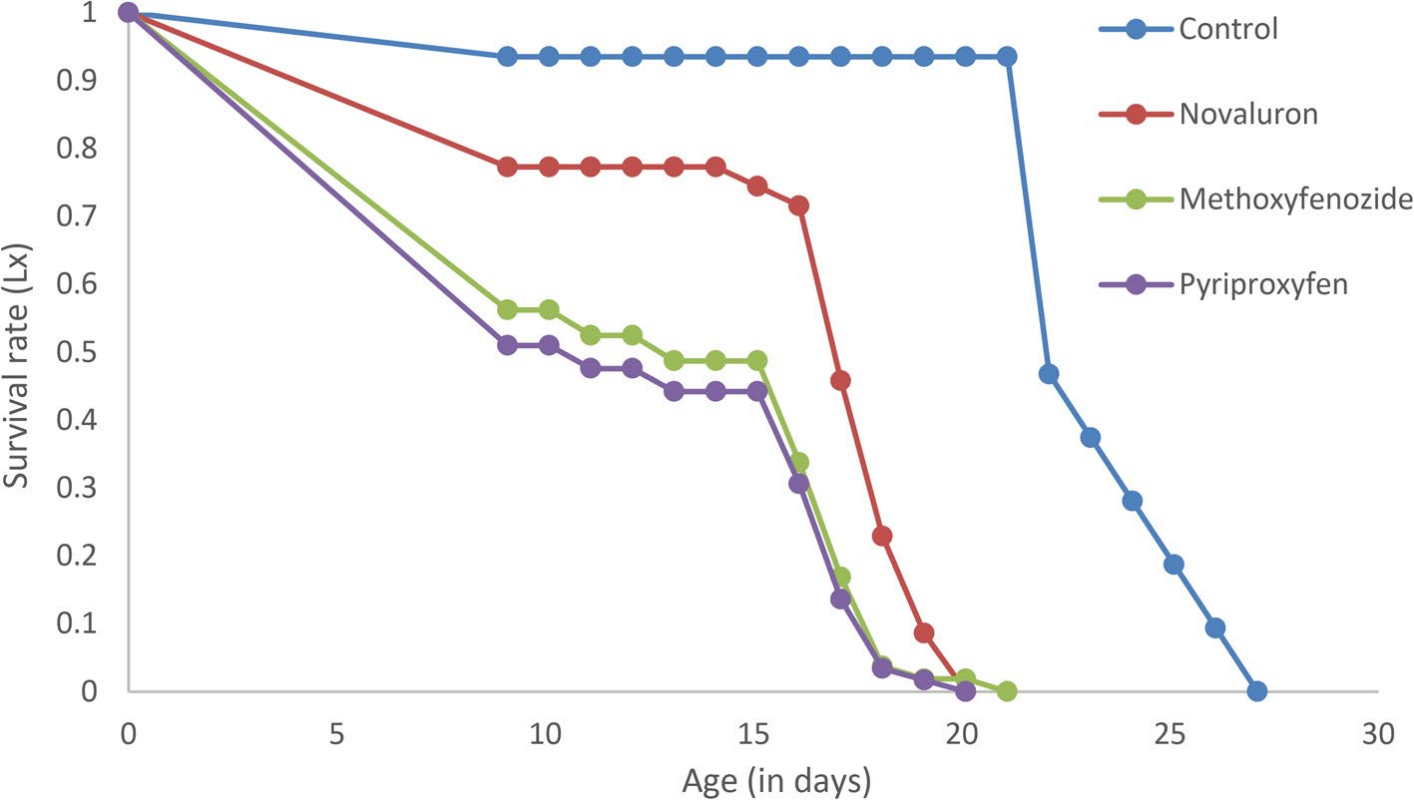
Survival Rate of H. hebetor Adults after IGR Exposure. Survival rate (L_x_) of *H. hebetor* after exposure of adults to LC_50_ values of IGRs for 24 h.

The observed survivorship curve (Fig. 2) of *H. hebetor* followed the type I pattern as described by Slobodkin (1980), with the control group maintaining a high survival rate until late in life, while the treated groups exhibited a marked decline in survival earlier. Notably, pyriproxyfen-treated adults showed the steepest decline, with a significant reduction in survival rate beginning around day 5 and reaching zero by day 18. Methoxyfenozide and novaluron treatments also reduced survival rates, with significant drops observed around days 10 and 15, respectively. In contrast, the control group maintained a high survival rate up to day 25, indicating the impact of IGRs on *H. hebetor* longevity.

**Fig. 3:**
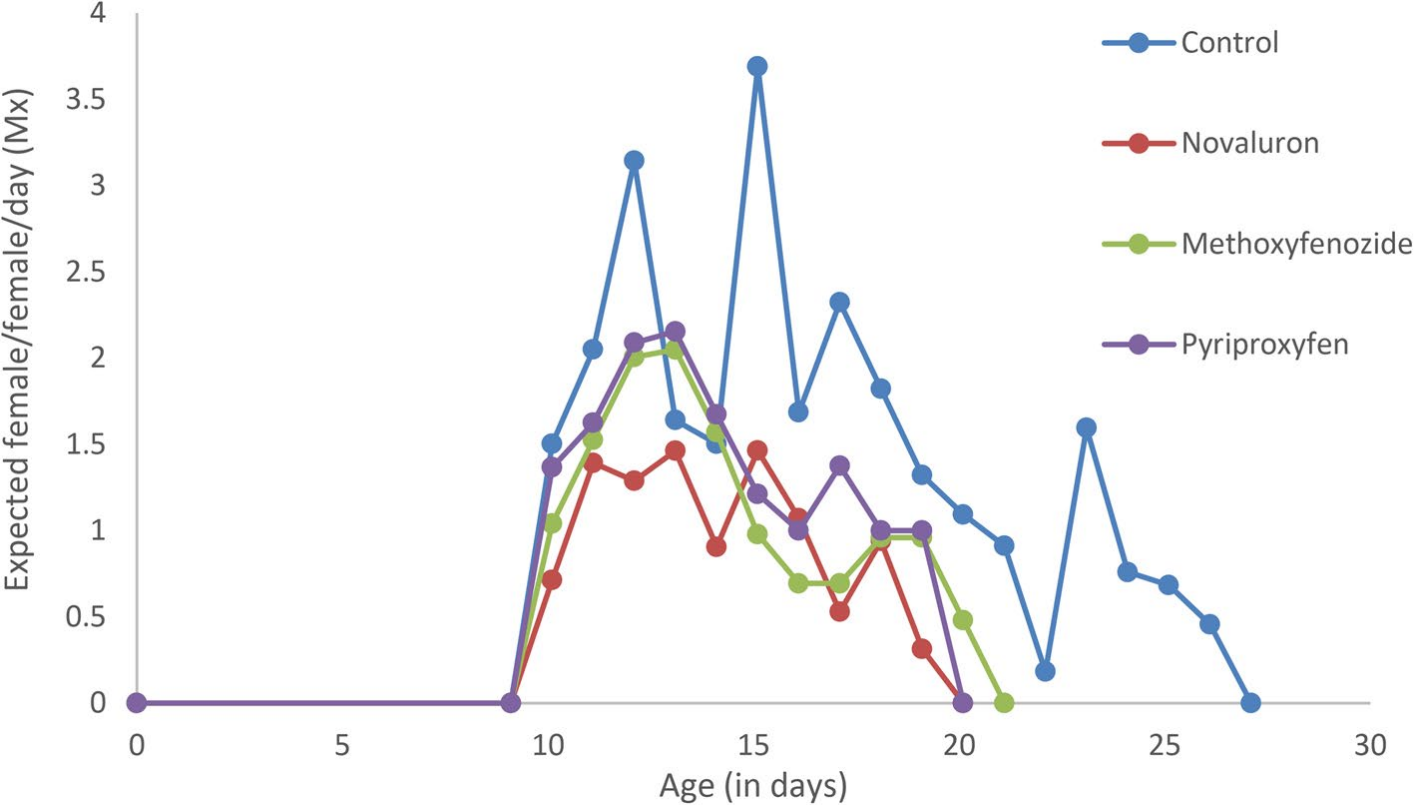
Daily Female Progeny Production of H. hebetor after IGR Treatment. Daily female progeny production (m_x_) of *H. hebetor* after treatment of adults with LC_50_ values of IGRs.

In terms of reproductive output, the daily female progeny production (m_x_) of *H. hebetor* was markedly influenced by the IGR treatments (Fig. 3). The highest peaks in m_x_ were observed in the control group, reaching up to 3.8 females per female per day around day 12. This peak was significantly higher than those observed in the treated groups. For the novaluron treatment, the peak in m_x_ was observed around day 11, but it was lower than the control, at approximately 2.1 females per female per day. Methoxyfenozide and pyriproxyfen treatments showed their highest m_x_ peaks at around day 10 and day 9, respectively, but these peaks were considerably lower than both the control and novaluron, demonstrating a suppressive effect on reproduction. The decline in m_x_ values occurred more rapidly in the IGR-treated groups compared to the control, highlighting the sublethal effects of these treatments on reproductive parameters.

## Discussion

Here, the tested IGRs did not affect the rates of paralysis and parasitism of *H. hebetor* nor did they affect the sex ratio of its offspring; however, hatching rates and immature *H. hebetor* survival rates were slightly reduced by IGRs. The longevity and fecundity of females in treated groups were each reduced to < 50% of their respective values in untreated females. According to International Organization for Biological Control recommendations, the effects of insecticides on nontarget organisms are categorized according to the reduction in beneficial capacity (i.e., parasitism) as harmful (> 99%), moderately harmful (80–99%), slightly harmful (50–79%), and harmless (< 50%)^43^. In the present study, the LC_50_ values of the tested IGRs caused < 50% reduction in the beneficial capacity of *H. hebetor*; thus, the IGRs can be considered harmless to this parasitoid species.

The data from the present study on the mild toxicity of IGRs on *H. hebetor* was consistent with previous findings indicating that IGRs are generally compatible with natural enemies and can be integrated into IPM programs. Chappell, et al.^44^, found that novaluron had minimal impact on beneficial arthropods in cotton fields, unlike imidacloprid and thiamethoxam, which significantly reduced predator populations. Similarly, Hernández, et al.^45^ reported that IGRs exhibited the least lethal effects on the parasitoids *Neochrysocharis formosa* (Westwood) (Hymenoptera: Eulophidae) and *Ganaspidium nigrimanus* (Kieffer) (Hymenoptera: Figitidae), affecting their survival rates and fecundity less than other insecticides. Suarez-Lopez, et al.^34^ demonstrated that combining microbial agents with IGRs could enhance larval mortality rates of *Spodoptera littoralis* (Boisduval) (Lepidoptera: Noctuidae), while maintaining compatibility with natural enemies. Ayalew^46^ concluded that novaluron could be used in IPM against diamondback moths because of its relatively limited effects against the associated parasitoids. Liu and Stansly^47^ and Ishaaya, et al.^48^ also found that pyriproxyfen and novaluron were relatively harmless to various *Encarsia* (Hymenoptera: Aphelinidae) species. In addition, Khan, et al.^49^ and Abedi, et al.^30^ found methoxyfenozide had minimal effects on *H. hebetor*’s survival, fecundity and functional response to different host densities. Rugno, et al.^50^ observed that it did not affect the duration and survival of the immature stages, but significantly reduced fecundity and longevity of the predator *Ceraeochrysa cincta* (Schneider) (Neuroptera: Chrysopidae), whereas pyriproxyfen reduced the larval survival by 19.5%, but did not affect the development, survival and reproduction of surviving individuals. Amarasekare, et al.^51^ observed that the parasitoid; *Trioxys pallidus* (Haliday) (Hymenoptera: Braconidae) had similar longevities to females in the control treatment. Recently, de Paiva, et al.^52^ considered methoxyfenozide as a selective insecticide for *Trichogramma pretiosum* Riley and they recommended its use in IPM programs due to its minimal impact on the pupal stage and parasitism capacity.

The current findings indicated that, of the tested IGRs, pyriproxyfen was the most toxic against *P. unionalis* larvae and had the most severe effects on the survival of immature *H. hebetor*. These results reflect the properties of pyriproxyfen, which directly affects metamorphosis and embryogenesis^21^. The effects may also be due to immature feeding disruption, as previously reported by Planes, et al.^53^ and Barbosa, et al.^54^ for immature coccinellid predators (*Cryptolaemus montrouzieri* Mulsant and *Tenuisvalvae notata* Muslant). He, et al.^55^ provided an explanation for these effects in relation to *Coccinella septempunctata* (Coleoptera: Coccinellidae) where he reported that the larvae directed a large amount of its metabolic energy to detoxify pyriproxyfen, which resulted in retarded larval growth and development. Similarly, Iftikhar, et al.^21^ noticed effects of pyriproxyfen on the fertility and development of various growth stages of the predatory ladybird; *Hippodamia convergens* Guerin–Meneville (Coleoptera: Coccinellidae); however, the sex ratio of this species’ progeny was not altered significantly by the IGRs relative to the sex ratio of the control group. Consistent with these findings, Saber and Abedi^39^ reported that the sex ratio of *H. hebetor* treated with LC_30_ levels of methoxyfenozide was 0.50 while that of the control was 0.55.

Several studies have emphasized the relevance of using factitious hosts to better understand the impacts of pesticides under controlled conditions, which can provide insights applicable to field conditions. For instance, Gill, et al.^56^ found significant reductions in parasitism and adult emergence rates of *Trichogramma chiloto nis* Ishii when using the factitious host *Corcyra cephalonica* (Stainton). In our study, *G. mellonella* was used to evaluate pesticide impacts on *H. hebetor* due to its availability and ease of rearing in laboratory conditions compared to the target pest. This method aligns with previous research that has successfully utilized factitious hosts to evaluate pesticide impacts. Factitious hosts standardize experiments and ensure reproducibility, which is crucial for accurately assessing pesticide effects on beneficial insects. Additionally, the use of factitious hosts allows researchers to study pesticide effects on biocontrol agents without risking the populations of natural hosts^57^.

Demographic toxicology is an approach by which the ecological impact of a toxicant on a population can be assessed. It is considered an important tool for the accurate assessment of various pesticide effects^58^. Generally, life table parameters are considered to be important population parameters that reflect the combined effects of biological attributes like developmental time, survival rates, reproductive capacity, and sex ratio^59^. Pesticide-induced reductions in demographic parameters can negatively affect the fecundity and survival of insects and consequently reduce population size in successive generations as well as limit the efficiency of pest control^60^. Many authors^61,62^ have recommended that r_m_ be used to assess overall pesticide effects because it is based on both fecundity parameters and survivorship. The r_m_ value determines the exponential increase of a population, where r_m_ > 0 indicates population increase, r_m_ = 0 indicates a constant population, and r_m_ < 0 indicates that a population is declining to extinction^63^. In the present study, r_m_ > 0 (0.19 day^−1^ for the control and 0.12–0.13 day^−1^ for IGR-treated insects), which indicates that *H. hebetor* treated with the tested IGRs exhibited exponential population increase. This is consistent with the findings of Ou, et al.^59^, who found that the r_m_ value above zero favours the population growth of the parasitoid. Nevertheless, the lower r_m_ in the treated population may influence subsequent generations^64^. Similar to the present output, Amarasekare, et al.^51^ found that Novaluron had no significant negative effects on the parasitoid *T. pallidus* and the estimated values of r_m_ remained positive. The r_m_ value is used to determine 𝜆; therefore, changes in 𝜆 values show the same patterns as changes in r_m_ values. The tested IGRs also resulted in lower mean T values, which could be considered an advantage for *H. hebetor* because low T values lead to an increased number of generations through the extended life cycle of the host. Additionally, Mahdavi, et al.^23^ found that insecticides have harmful effects on parasitoids when they cause an increase in mean T. DT is another important population parameter; it indicates when a population will double in size. DT was slightly affected by the IGRs in the current study. In contrast, Iftikhar, et al.^21^ reported noticeable effects of pyriproxyfen on prolonged preadult developmental duration in the predatory ladybird *H. convergens*. Similar to the present results, Iftikhar, et al.^21^ also reported that the sublethal effects of pyriproxyfen reduced the population growth parameters of *H. convergens*.

By plotting survivorship against time, the influence of mortality on a population can often be determined. Type I populations were found during the present work, which indicates that mortality mostly occurred in older-aged insects. Similarly, Mahdavi, et al.^23^ found that *H. hebetor* populations followed a type I curve after treatment with both carbaryl and abamectin. The observed leftward shift of the curve following IGR treatments indicated that their harmful effects coincided with those recorded for the same parasitoid after treatment with both azadirachtin and cypermethrin^65^. Conversely, trends in m_x_ curves over the lifespan of females showed alternate decreases and increases in female emergences that were clearly reduced due to treatment.

In summary, the findings of the present study reflect the compatibility of the IGRs novaluron, methoxyfenozide, and pyriproxyfen with the larval parasitoid *H. hebetor*, which is associated with the olive leaf moth. The IGRs could be considered harmless because they did not substantially affect the beneficial capacities of the parasitoids. In addition, *H. hebetor* populations showed a tendency to increase under IGR treatment, as shown by r_m_ values, although some slight adverse effects were observed. The current findings align with the literature which emphasizes the importance of understanding both lethal and sublethal effects, such as altered behaviour and impaired development, which significantly influence the ecological roles of beneficial insects^25^. Further studies^21,22^ have demonstrated that sublethal doses of pesticides can impair the growth and reproductive capabilities of natural enemies, thereby reducing their efficacy in IPM programs. Thus, future studies should be focused on the potential long-term effects of IGRs, which could reveal possible decreases in parasitoid biological activity due to transgenerational effects of the chemicals on demography and parasitism. Although the present experiments were carried out under laboratory conditions, the results obtained could be helpful to evaluate efficacy of *H. hebetor* in natural conditions^66^ when integrated with the studied IGRS.

These results should also be confirmed using field studies to validate the safety of the tested IGRs. Finally, it will be important to study the broader effects of IGRs on other beneficial insects and expand the evaluation of their impacts to the community and/or ecosystem levels. Such studies might encourage the wide application of IGRs, either alone or integrated with the field release of parasitoids, to combat the olive leaf moth, *P. unionalis*, in olive orchards.

## Data Availability

The datasets generated during and/or analysed during the current study are available from the corresponding author on reasonable request.
